# Supplementary material for: Flame-made ternary Pd-In2O3-ZrO2 catalyst with enhanced oxygen vacancy generation for CO2 hydrogenation to methanol
Source: Nat Commun. 2022 Sep 24;13:5610. doi: 10.1038/s41467-022-33391-w (PMC9509363; doi:10.1038/s41467-022-33391-w)
Supplement: Supplementary file 1 — Supplementary Information [file 41467_2022_33391_MOESM1_ESM.pdf]

Supplementary Information

Flame-made ternary Pd-In_2O_3-ZrO_2 catalyst with enhanced oxygen vacancy generation for CO_2 hydrogenation to methanol

Pinheiro Araújo *et al.*

Table of Contents

Supplementary Methods 2
Additional Details on Catalyst Preparation 2
Supplementary Discussion 4
Origin of Broad Ferromagnetic Signals in EPR Spectra of In_2O_3-Based Catalysts 4
Insights into Palladium Speciation by EPR Spectroscopy 4
Supplementary Tables 1-6 6
Supplementary Figures 1-18 12
Supplementary References 31

Supplementary Methods

Additional Details on Catalyst Preparation

Tetragonal zirconias (t-ZrO_2) were synthesized through precipitation methods using distinct precipitating agents, including ammonium hydroxide,[1] sodium hydroxide,[2,3] and ethylenediamine.[1] Briefly, 15 g of a $ZrO(NO_3)_2$ solution (Sigma-Aldrich, 35 wt.% in diluted HNO_3, >99% trace metals basis) was diluted with deionized water (300 cm^3). Ethylenediamine ($Et(NH_2)_2$, Sigma-Aldrich, >98%), NH_4OH (25 wt.%, Merck), or aqueous NaOH (5 M, Sigma Aldrich, 98%) were added dropwise (*ca.* 3 cm^3 min^{-1}) to the solution until reaching pH 9.2 and the resulting slurry was stirred at 353 K for 3 h. The precipitate was recovered by high-pressure filtration, washed three times with deionized water (1 dm^3 each time), dried in a vacuum oven (2 kPa, 323 K, 12 h), and calcined in static air at 973 and 773 K (3 K min^{-1}) for 3 h, when the amine and the inorganic hydroxides were used, respectively.

Supported catalysts (**5In_2O_3/*t*-ZrO_2,WI** and **5In_2O_3/*m*-ZrO_2,WI**) containing 5 wt.% In_2O_3 were prepared by wet impregnation (WI) using the precipitated t-ZrO_2 prepared and commercial *monoclini*c zirconia (m-ZrO_2, Saint-Gobain NorPro, 95%), respectively.[1] WI encompassed suspending 2 g of support in a mixture of deionized water (54 cm^3), ethanol (70 cm^3, Merck, 99%), and $In(NO_3)_3 \cdot 6H_2O$ (0.2776 g, Merck, 99%). The resulting slurry was magnetically stirred (800 rpm) at room temperature for 12 h. Thereafter, the solvent was removed using a rotary evaporator (Büchi Rotavap R-114) at 323 K, keeping the slurry constantly at boiling point by lowering the pressure from 180 to 40 mbar. The solid was then dried in a vacuum oven (2 kPa, 323 K, 12 h) and calcined in static air at 773 K (2 K min^{-1}) for 3 h.

5In_2O_3-ZrO_2,CP was synthesized through co-precipitation (CP). Following a reported method,[1] 15 g of a $ZrO(NO_3)_2$ solution (Sigma-Aldrich, 35 wt.% in diluted HNO_3, >99% trace metals basis) and 0.4139 g of $In(NO_3)_3 \cdot 6H_2O$ (Merck, 99%) were diluted with deionized water (300 cm^3). NH_4OH (25 wt.%, Merck) was added dropwise (*ca.* 3 cm^3 min^{-1}) to this solution until reaching pH 9.2 and the resulting slurry was stirred at 353 K for 3 h. The precipitate was

recovered by high-pressure filtration, washed three times with deionized water (1 dm^3 each time), dried in a vacuum oven (2 kPa, 323 K, 12 h), and calcined in static air at 773 K (2 K min^{-1}) for 3 h.

Pd-In_2O_3,CP containing 0.75 wt.% Pd was produced by coprecipitation.[4] In this case, $In(NO_3)_3 \cdot 7H_2O$ (3.5 g) and $Pd(NO_3)_2 \cdot 5H_2O$ (0.025 g, Sigma-Aldrich) were dissolved in deionized water (50 cm^3). A solution of Na_2CO_3 (10 g) in deionized water (100 cm^3) was added dropwise under magnetic stirring (500 rpm) until pH 9.2. The resulting slurry was aged stirring at 800 rpm and room temperature for 1 h. Thereafter, it was diluted with additional deionized water (50 cm^3) and the precipitate was recovered by high-pressure filtration, washed three times with deionized water (2 dm^3 each time), dried in a vacuum oven (2 kPa, 323 K, 12 h), and calcined in static air at 573 K (2 K min^{-1}) for 3 h.

Pd-In_2O_3/*m*-ZrO_2,WI, containing 5 wt.% In_2O_3 and 0.75 wt.% Pd, was prepared by wet co-impregnation of indium and palladium on *monoclinic* zirconia. Briefly, 5.2 g of *m*-ZrO_2 (Saint-Gobain NorPro, 95%) were suspended in a solution of 0.6954 g of $In(NO_3)_3 \cdot 5H_2O$ (Alfa Aesar, 99.999%) and 0.01989 g of $Pd(NO_3)_3 \cdot 11H_2O$ (Sigma Aldrich, 99.999% metal basis) in deionized water (124 cm^3). The resulting slurry was magnetically stirred (500 rpm) at room temperature for 12 h. Thereafter, the solvent was removed using a rotary evaporator (Büchi Rotavap R-114) at 323 K, keeping the slurry constantly at boiling point by lowering the pressure from 180 to 40 mbar. The solid was then dried in a vacuum oven (2 kPa, 323 K, 12 h) and calcined in static air at 773 K (2 K min^{-1}) for 3 h.

Supplementary Discussion

Origin of broad ferromagnetic signals in EPR spectra of In_2O_3-based catalysts

The broadest signals (*ca.* 5 kG broad, **Figure 7b**), associated with the presence of oxygen vacancies are more clearly visible around room temperature and are often undetectable at low temperature because of further broadening. This feature, together with the hysteretic behaviour consisting of a difference in intensity and lineshape for upwards and downwards field sweeps (**Supplementary Fig. 16**), indicate ferromagnetic behaviour. These signals can therefore be attributed to strongly interacting paramagnetic centres. For In_2O_3 and other metal oxides, this unexpected behaviour is attributed to long-range exchange coupling between spins, due to oxygen vacancies. At low concentration, vacancies introduce intra-bandgap energy states, where unpaired electrons can be trapped (colour centres), giving rise to sharp and nearly isotropic EPR signals (as those observed for In_2O_3/*t*-ZrO_2,WI) with a *g* factor close to the free electron value. When vacancy concentration is sufficiently high, an impurity band is formed. If the density of states near the Fermi energy is large enough (Stoner criterion), the spin-up and spin-down states split spontaneously, resulting in a ferromagnetic behaviour.[5] When empty or partially filled *d* orbitals overlap with the impurity band, electron hopping may occur, giving rise to long-range exchange interactions and consequently very high Curie temperatures, which often allow for the observation of ferromagnetism even at room temperature.[6] The broad signals observed for most of the analyzed samples can therefore be attributed to oxygen vacancy-related ferromagnetism, which appears at high vacancy concentrations.

Insights into palladium speciation by EPR spectroscopy

The fresh Pd-In_2O_3, Pd-ZrO_2, and Pd-In_2O_3-ZrO_2 samples display additional signals (**Figure 7a,b**), with very different characteristics compared to V_o, Zr^{3+}, and O_2^- signals. They are significantly broader and their *g* tensors are highly anisotropic. These features are related to strong spin-orbit coupling and the signals can be therefore attributed to Pd-centred paramagnetic species. Pd-In_2O_3-ZrO_2 shows a signal with a well-resolved axial *g* anisotropy

(g_{xx} = g_{yy} = 2.33, g_{zz} = 2.07), most likely due to isolated Pd^{3+} ions substituting In^{3+} ions in the In_2O_3 lattice. This interpretation, based on the relative magnitude of the principal values of the *g* tensor, is confirmed by the fact that no such signal is observed in the reference Pd-ZrO_2 sample. Indeed, the latter only shows a weak, broad signal with poorly resolved *g* anisotropy centred around *g* = 2, emerging after the reaction. This signal is most probably due to small Pd clusters, which notably form by aggregation on the surface of the support during CO_2 hydrogenation. Such clusters often show a non-zero magnetic moment for nm-size clusters and particles.[7] In this case, Pd is therefore mostly located on the surface of ZrO_2.

Supplementary Tables

Supplementary Table 1. Synthesis parameters of the catalysts prepared by flame spray pyrolysis (FSP).

Catalyst	Metal concentration in precursor solution (mol $kg_{solution}^{-1}$)		
	Pd or Pt[a]	In[b]	Zr[c]
In_2O_3	-	0.43	-
$5In_2O_3$-ZrO_2	-	$2.03\cdot10^{-2}$	0.46
0.75Pd-ZrO_2	$4.30\cdot10^{-3}$	-	0.49
0.75Pd-In_2O_3	$4.27\cdot10^{-3}$	0.43	-
0.5Pd-$5In_2O_3$-ZrO_2	$3.13\cdot10^{-3}$	$2.38\cdot10^{-2}$	0.51
0.75Pd-$5In_2O_3$-ZrO_2	$4.65\cdot10^{--3}$	$2.37\cdot10^{-2}$	0.50
1Pd-$5In_2O_3$-ZrO_2	$5.40\cdot10^{-3}$	$2.08\cdot10^{-2}$	0.46
1.5Pd-$5In_2O_3$-ZrO_2	$9.04\cdot10^{-3}$	$2.29\cdot10^{-2}$	0.49
2Pd-$5In_2O_3$-ZrO_2	$1.19\cdot10^{-2}$	$2.31\cdot10^{-2}$	0.48
1Pt-In_2O_3	$3.07\cdot10^{-3}$	0.43	-
1Pt-$5In_2O_3$-ZrO_2	$3.33\cdot10^{-3}$	$2.29\cdot10^{-2}$	0.48

[a] Palladium and platinum were directly added to the precursor solutions as $Pd(acac)_2$ (ABCR, 99%) and $Pt(acac)_2$ (ABCR, 99%), respectively, and ultrasonicated at room temperature for 10 min. [b] An indium precursor stock solution was prepared by dissolving $In(OAc)_3$ (99.3 g, Sigma-Aldrich, 99.999% trace metal basis) with 2-ethylhexanoic acid (500 g, Sigma-Aldrich, 99%) at 433 K for 5 h. The resulting solution was distilled under reduced pressure for 30 min to a total weight of 540 g and diluted with tetrahydrofuran (THF, Sigma-Aldrich, LiChrosolv, 99.9%) to a total weight of 800 g. This corresponds to a metal weight content of 4.9 wt.% In. [c] A zirconium precursor stock solution was prepared by dilution of zirconium 2-ethylhexanoate (250 g, ABCR, 6% Zr in mineral spirit) with a mixture of 2-ethylhexanoic acid and THF (2:1, w/w) to a total metal weight concentration of 5.0 wt.% Zr.

Supplementary Table 2. Bulk composition of the catalysts investigated.

Catalyst	Bulk content (wt.%)			
	Pd or Pt		In_2O_3	
	Nominal	Measured	Nominal	Measured
In_2O_3	-	-	-	-
$5In_2O_3$-ZrO_2	-	-	5	5.01[a]
$5In_2O_3$/*t*-ZrO_2,WI	-	-	5	5.54[a]
$5In_2O_3$/*t*-ZrO_2,WI-B1	-	-	5	4.98[a]
$5In_2O_3$/*t*-ZrO_2,WI-B2	-	-	5	5.78[a]
$5In_2O_3$/*m*-ZrO_2,WI	-	-	5	5.74[a]
$5In_2O_3$-*t*-ZrO_2,CP	-	-	5	4.78[a]
0.75Pd-ZrO_2	0.75	0.77[b]	-	-
0.75Pd-In_2O_3	0.75	0.61[b]	-	-
0.75Pd-In_2O_3,CP	0.75	0.85[b]	-	-
0.75Pd-$5In_2O_3$-ZrO_2,WI	0.75	0.95[b]	5	4.32[b]
0.75Pd-$5In_2O_3$-ZrO_2	0.75	0.81[b]	5	4.86[b]
0.5Pd-$5In_2O_3$-ZrO_2	0.5	0.52[b]	5	4.81[b]
1Pd-$5In_2O_3$-ZrO_2	1	1.03[b]	5	4.61[b]
1.5Pd-$5In_2O_3$-ZrO_2	1.5	1.66[b]	5	4.93[b]
2Pd-$5In_2O_3$-ZrO_2	2	2.19[b]	5	4.71[b]
1Pt-In_2O_3	1	1.05[a]	-	-
1Pt-$5In_2O_3$-ZrO_2	1	1.24[a]	5	5.21[a]

[a]XRF and [b]ICP-OES.

Supplementary Table 3. Comparative performance of heterogeneous catalysts under similar reaction conditions for CO_2 hydrogenation to methanol.

Catalyst	T	$GHSV$	X_{CO2}	S_{MeOH}	STY	STY
	(K)	($cm^3\ h^{-1}\ g_{cat}^{-1}$)	(%)	(%)	($g_{MeOH}\ h^{-1}\ g_{cat}^{-1}$)	($g_{MeOH}\ h^{-1}\ g_{In}^{-1}$)
Pd-In_2O_3-ZrO_2[a]	553	48,000	12	87	1.29	31.2
Pd-In_2O_3-ZrO_2[a]	553	24,000	16	77	0.73	17.6
PdIn/SiO_2[b]8	573	63,000	16	61	0.60	1.19
Cu-In-Zr-O[c]9	523	36,000	1.5	80	0.08	0.10
Pd-In_2O_3[4]	553	48,000	10	78	1.01	1.01
Pd-In_2O_3[4]	553	24,000	12	75	0.61	0.61
Pd-P/In_2O_3[10]	573	21,000	20	72	0.89	0.89
Rh-In_2O_3[11]	573	21,000	17	56	0.54	0.55
Pt-In_2O_3[12]	573	21,000	17	53	0.44	0.55
Au-In_2O_3[13]	573	21,000	12	68	0.47	0.47
Ni-In_2O_3[14]	573	21,000	19	54	0.55	0.56
In@Co-1[15]	573	19,200	19	69	0.48	1.16
In_2O_3/*m*-ZrO_2[10]1	553	24,000	4.5	84	0.25	4.44
In_2O_3/*t*-ZrO_2[1]	553	24,000	0.5	82	0.04	0.65
In_2O_3[1]	553	24,000	3.9	84	0.22	0.18
ZnO-ZrO_2[16]	593	24,000	10	86	0.71	-
Cu-ZnO-ZrO_2[d]17	513	55,000	9.7	62	1.20	-

[a] This work. P = 50 bar, except for [b] and [c], which are equal to 25 and 40 bars, respectively.H_2/CO_2 = 4, except for [d], which is equal to 3.

Supplementary Table 4. Results of the EXAFS spectra fitting of the FSP-prepared catalysts presented in **Figure 5c** with the uncertainty associated with the values.

Catalyst[a]	Scattering path	Δ*E* (eV)	Number of neighbors	*R* (Å)	Debye-Waller factor ($Å^2$)
0.75Pd-ZrO_2[b]	Pd-Pd	−5.7 ± 0.7	10.0 ± 1.1	2.80 ± 0.05	0.009 ± 0.001
0.75Pd-In_2O_3[c]	Pd-Pd(In)	−2.8 ± 1.4	8.6 ± 1.6	2.75 ± 0.00	0.017 ± 0.002
0.75Pd-5In_2O_3-ZrO_2[d]	Pd-Pd(In)	−7.9 ± 2.9	9.9 ± 3.8	2.68 ± 0.07	0.019 ± 0.006

[a] Catalysts after CO_2 hydrogenation for 20 h. Reaction conditions: $T = 553$ K, $P = 5$ MPa, $H_2/CO_2 = 4$, and $GHSV = 48{,}000$ cm^3 h^{-1} g_{cat}^{-1}. EXAFS spectra were fitted in the optimal *k*- ([b] 3-9.5, [c] 3-10, and [d] 3-8 $Å^{-1}$, respectively) and *R*-windows ([b-d] 1-3 Å). The Pd-Pd distance elongation (*R*) with respect to the R_{eff} (2.75 Å) evidenced for Pd-ZrO_2 indicates the formation of palladium hydride (PdH_x) upon reaction, which is in line with previous reports.[18-20]

Supplementary Table 5. Spectrometer settings applied for *ex situ* and *in situ* EPR experiments performed at distinct temperatures.[a]

T (K)	Microwave frequency (GHz)	Centre field (G)	Sweep width (G)	Modulation amplitude (G)	Conversion time (ms)	Time constant (ms)
20	9.4	3370	1000	1	327.68	81.92
303	9.8	3300	5900	5	655.36	163.84
553	9.2	3000	5900	3	655.36	163.84

[a] Modulation frequency, microwave power, and power attenuation were set to 100 kHz, 2.002 mW, and 20 dB, respectively.

Supplementary Table 6. Methanol space-time yield (*STY*) of selected catalysts presented in **Fig. 2** with the corresponding peak-to-peak linewidth measured by EPR.

Catalyst[a]	*STY* (g_{MeOH} h^{-1} g_{cat}^{-1})	Peak-to-peak linewidth (G)[b]	Relative [V_O] (-)[c]
$5In_2O_3$/*t*-ZrO_2,WI	0.00395	1037	0.31396
$5In_2O_3$-*t*-ZrO_2,CP	0.11828	1329	0.40236
$5In_2O_3$/*m*-ZrO_2,WI	0.2997	1320	0.39964
$5In_2O_3$-ZrO_2	0.35728	1556	0.47109
0.75Pd-$5In_2O_3$-ZrO_2[d]	1.32248	3303	1

[a] Catalysts after CO_2 hydrogenation for 50 h. Reaction conditions: $T = 553$ K, $P = 5$ MPa, $H_2/CO_2 = 4$, and $GHSV = 48{,}000$ cm^3 h^{-1} g_{cat}^{-1}. [b] Peak-to-peak linewidth were acquired from EPR spectra of used samples measured at 303 K and depicted in **Fig. 7b** and **Supplementary Fig. 18**. [c] Relative concentration of oxygen vacancies ([V_O]) was determined by applying a divide by max normalization to peak-to-peak linewidth values shown in [b].

Supplementary Figures

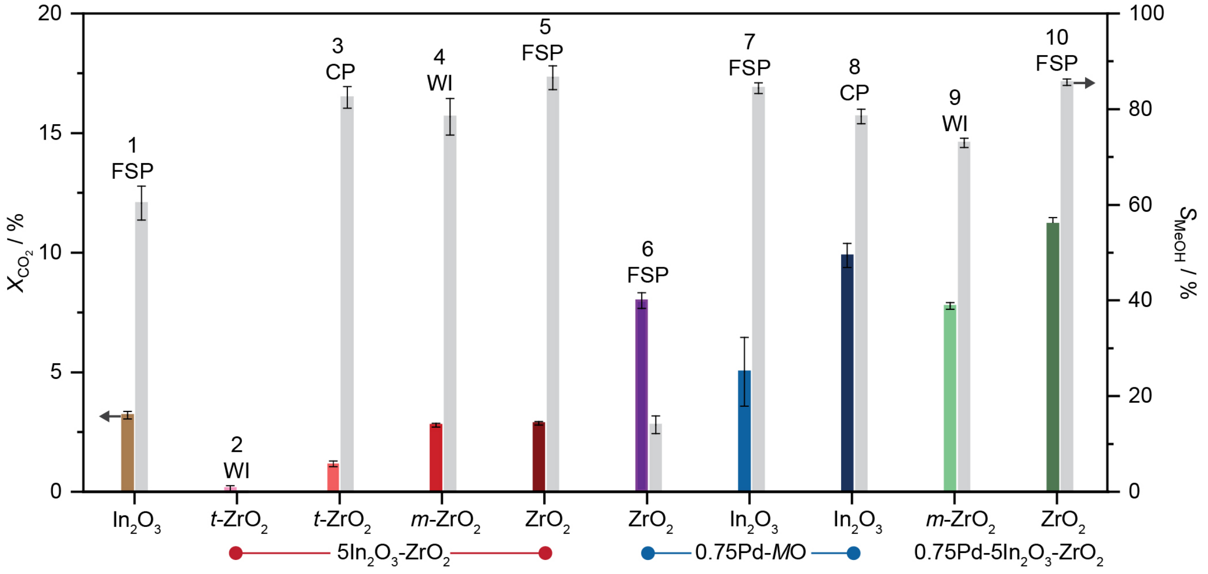

Supplementary Figure 1. Comparative CO_2 conversion and methanol selectivity of In_2O_3-based catalysts. CO_2 conversion (X_{CO2}, coloured bars) and methanol selectivity (S_{MeOH}, grey bars) over the ternary Pd-In_2O_3-ZrO_2 catalyst prepared by FSP-flame spray pyrolysis with benchmark In_2O_3-based catalysts and Pd-ZrO_2 serving as reference (WI-wet impregnation, and CP-coprecipitation). Averaged values measured during CO_2 hydrogenation over 50 h on stream are presented with their corresponding error bars. The number preceding the acronym in the catalyst codes indicates the nominal loading in wt.%, unless otherwise stated. Reaction conditions: T = 553 K, P = 5 MPa, H_2/CO_2 = 4, and $GHSV$ = 48,000 $cm^3\ h^{-1}\ g_{cat}^{-1}$.

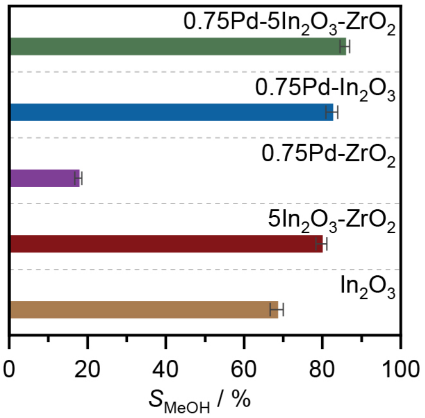

Supplementary Figure 2. Methanol selectivity at comparable CO_2 conversion levels. Methanol selectivity (S_{MeOH}) for CO_2 hydrogenation to methanol over selected catalysts prepared by FSP. S_{MeOH} was assessed at constant CO_2 conversion (*ca.* 3%), variable *GHSV* = 48,000-60,000 $cm^3\ h^{-1}\ g_{cat}^{-1}$, T = 553 K, P = 5 MPa, and H_2/CO_2 = 4.

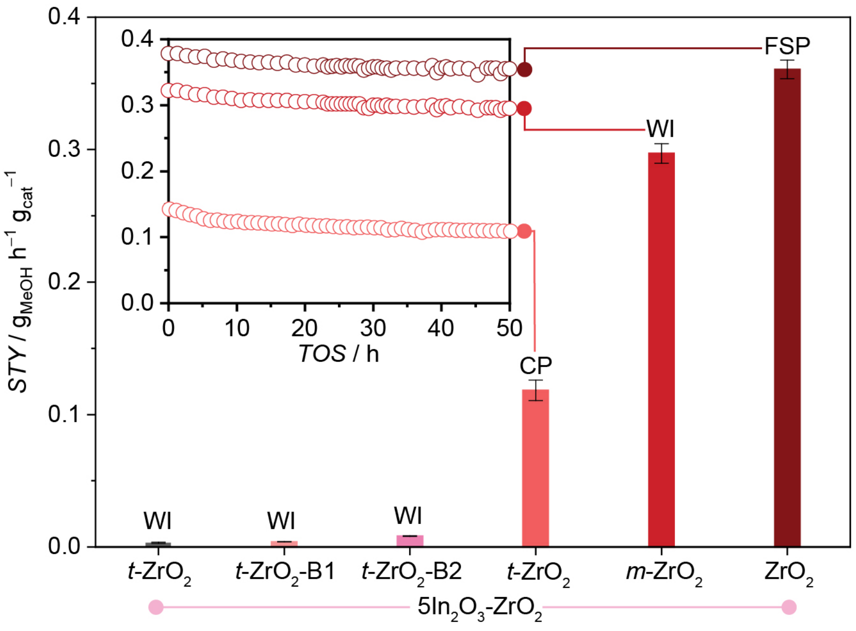

Supplementary Figure 3. Comparative methanol productivity of ZrO_2-supported In_2O_3 catalysts. Methanol space-time yield (*STY*) during CO_2 hydrogenation over ZrO_2-supported In_2O_3 catalysts prepared by distinct synthesis methods (FSP-flame spray pyrolysis, WI-wet impregnation, and CP-coprecipitation). B1 and B2 stand for In_2O_3 wet impregnated onto *t*-ZrO_2 attained by precipitation using a base different from NH_4OH (NaOH and ethylenediamine, respectively). Averaged values measured during CO_2 hydrogenation over 50 h on stream are presented with their corresponding error bars. The nominal loading of In_2O_3 is 5 wt.%. The inset shows the methanol *STY* of selected systems over time-on-stream, *TOS*, evidencing their stable performance. Reaction conditions: $T = 553$ K, $P = 5$ MPa, $H_2/CO_2 = 4$, and $GHSV = 48{,}000$ cm^3 h^{-1} g$_{cat}{}^{-1}$. In_2O_3/*t*-ZrO_2 catalysts prepared by WI and CP display mediocre methanol productivity, indicating that these synthesis methods, as opposed to FSP, are not suitable to attain effective *t*-ZrO_2-supported In_2O_3 catalysts.

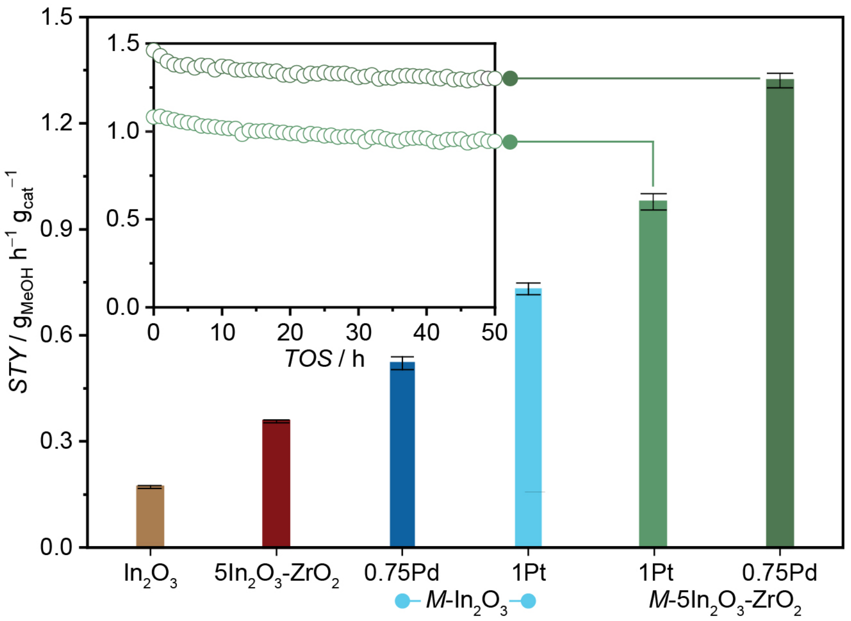

Supplementary Figure 4. Comparative methanol productivity of platinum-promoted catalysts. Methanol space-time yield (*STY*) during CO_2 hydrogenation over In_2O_3-based catalysts prepared by FSP. Averaged values measured during CO_2 hydrogenation over 50 h on stream are presented with their corresponding error bars. Inset shows methanol *STY* of ternary Pd- and Pt-In_2O_3-ZrO_2 systems over time-on-stream, *TOS*, evidencing their stable performance. Reaction conditions: $T = 553$ K, $P = 5$ MPa, $H_2/CO_2 = 4$, and $GHSV = 48{,}000$ cm^3 h^{-1} g_{cat}^{-1}. Similar to the Pd-containing ternary catalyst, Pt-In_2O_3-ZrO_2 also shows a superior performance in comparison to its binary counterpart, highlighting that FSP is a versatile method to produce *M*-In_2O_3-ZrO_2 catalysts with boosted methanol productivity.

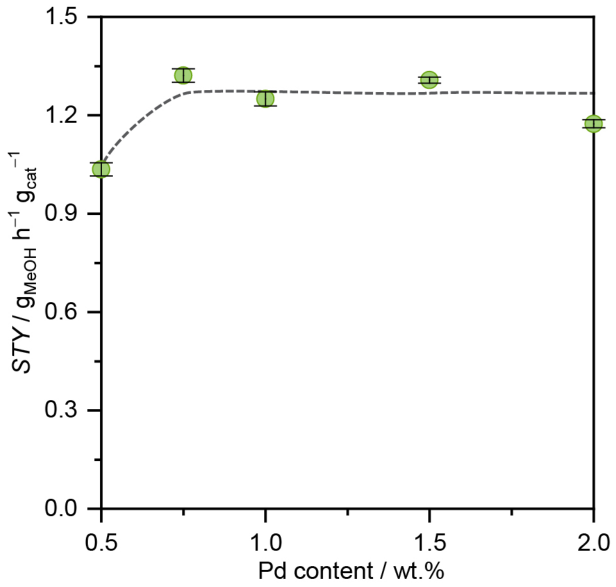

Supplementary Figure 5. Impact of promoter content on the performance of ternary Pd-In_2O_3-ZrO_2 catalysts. Space-time yield (*STY*) as a function of promoter content during CO_2 hydrogenation over Pd-In_2O_3-ZrO_2 catalysts prepared by FSP. Averaged values measured during CO_2 hydrogenation over 50 h on stream are presented with their corresponding error bars. Reaction conditions: $T = 553$ K, $P = 5$ MPa, $H_2/CO_2 = 4$, and $GHSV = 48{,}000$ cm^3 h^{-1} g_{cat}^{-1}. No further improvement in methanol productivity is attained by increasing the Pd loading beyond 0.75 wt.%, most likely owing to sintering of palladium at higher loadings.

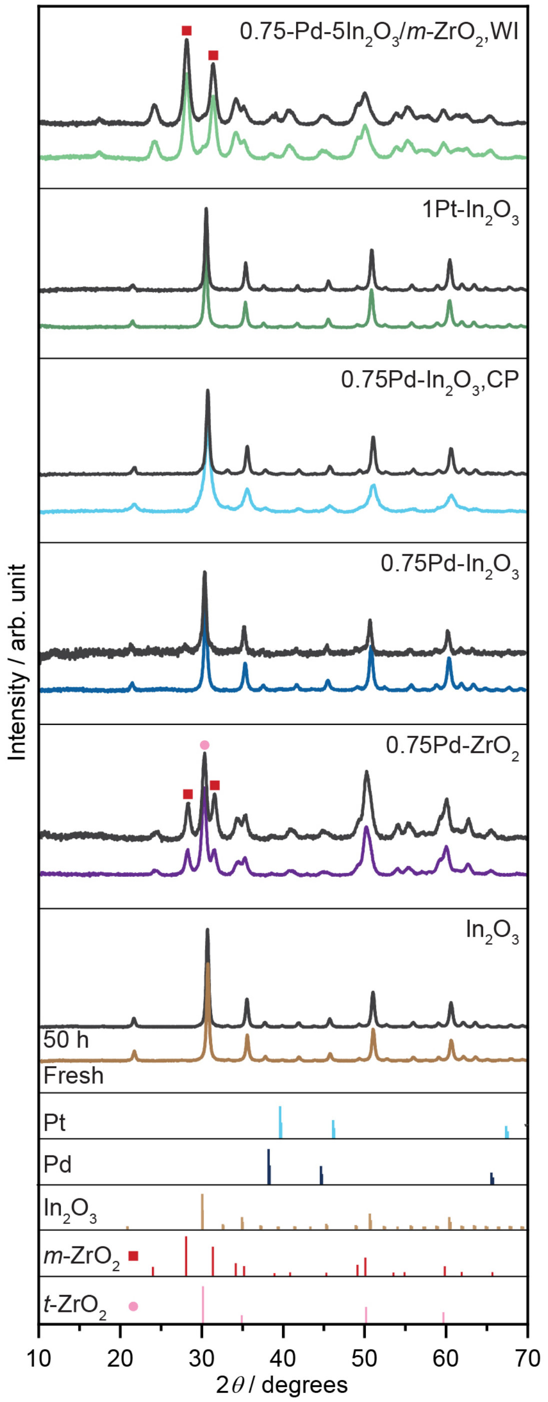

Supplementary Figure 6. Bulk structure of In_2O_3-based catalysts. XRD patterns of In_2O_3-based and Pd-ZrO_2 catalysts in fresh form and after CO_2 hydrogenation for 50 h. Reference patterns of pure phases are shown with vertical lines in the bottom panel. No reflections characteristic of promoters in the metallic state indicating sintering were detected, discarding significant sintering upon reaction.

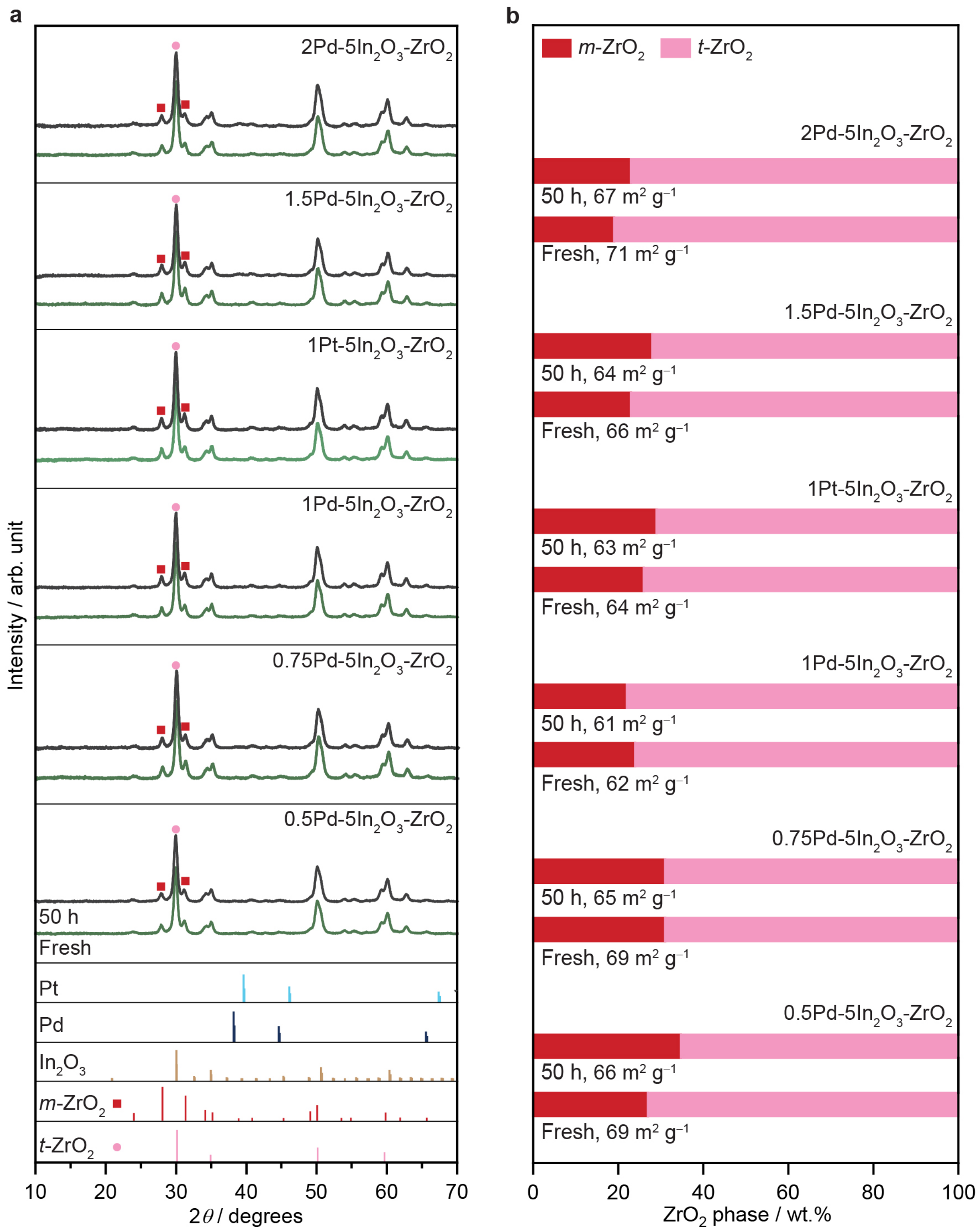

Supplementary Figure 7. Bulk structure of ternary Pd-In_2O_3-ZrO_2 catalysts. (**a**) XRD patterns and (**b**) ZrO_2 phase composition and BET surface areas (S_{BET}), of Pd- and Pt-In_2O_3-ZrO_2 catalysts prepared by FSP in fresh form and after CO_2 hydrogenation for 50 h. Reference XRD patterns of pure phases are shown with vertical lines in **a** at the bottom panel. Phase composition shown in **b** was determined from the XRD patterns presented in **a** by applying the reference intensity ratio method, whereas S_{BET} was determined using the N_2 isotherm. *Tetragonal* and *monoclinic* zirconia (*ca.* 70-80 wt.% *versus* 20-30 wt.%, respectively) are present in all ternary catalysts prepared by FSP.

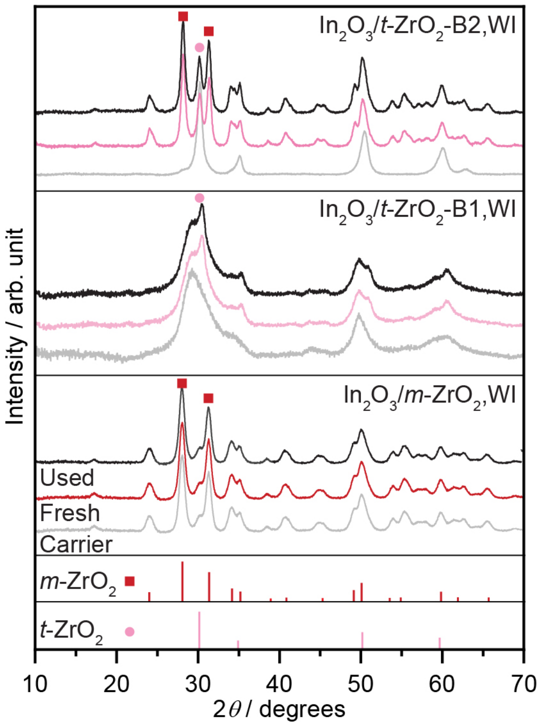

Supplementary Figure 8. Bulk structure of ZrO_2-supported In_2O_3 catalysts. XRD patterns of zirconia-supported In_2O_3 catalysts prepared by WI-wet impregnation in fresh form and after CO_2 hydrogenation for 50 h. B1 and B2 stand for In_2O_3 wet impregnated onto *t*-ZrO_2 attained by precipitation using different bases (ethylenediamine and NaOH, respectively). Reference patterns of pure phases are shown with vertical lines at the bottom panel. The *t*-ZrO_2 carrier partially transforms into *m*-ZrO_2 upon deposition of In_2O_3, which is less evident for In_2O_3/*t*-ZrO_2-B1,WI due to the broader reflections in this sample.

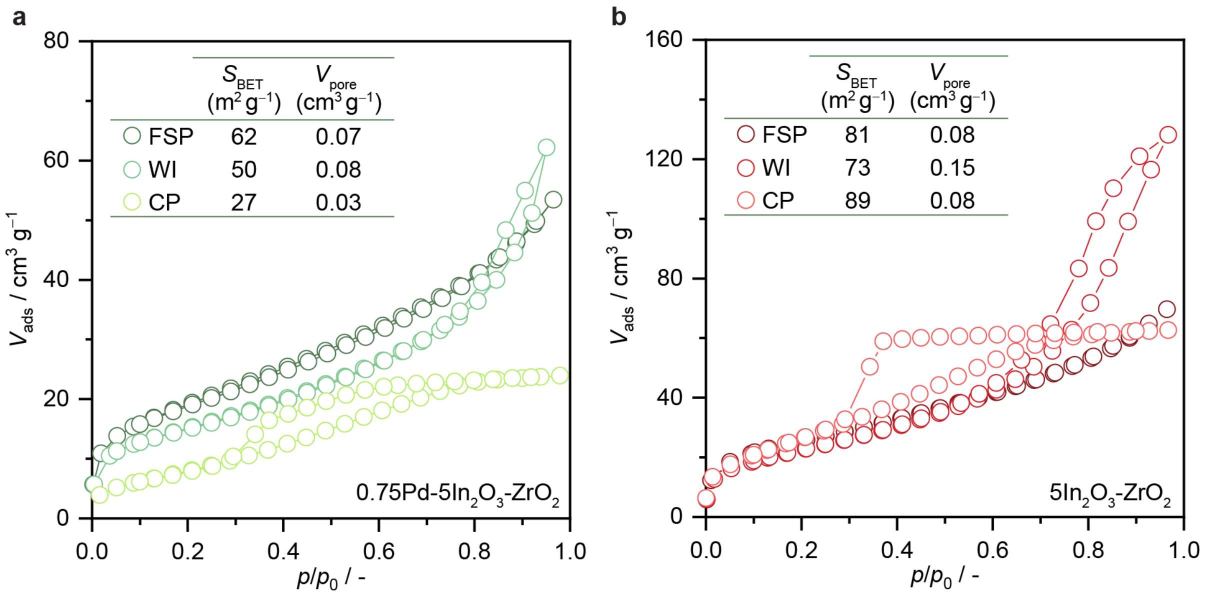

Supplementary Figure 9. Textural properties of In_2O_3-based catalysts. Gas sorption isotherms collected at 77 K, with corresponding BET surface areas (S_{BET}) and pore volumes (V_{pore}) shown in the table inset, of (**a**) ternary Pd-In_2O_3-ZrO_2 and (**b**) binary In_2O_3-ZrO_2 catalysts in fresh form prepared by WI-wet impregnation, CP-co-precipitation, and FSP-flame spray pyrolysis. All systems exhibit similar textural properties, except for Pd-In_2O_3-ZrO_2, CP. The latter displays an inferior surface area compared to its counterparts, further confirming that CP is not an ideal synthesis method to produce Pd-In_2O_3-ZrO_2 catalysts with superior performance. Hence, this sample was considered unsuitable for a fair comparison with the other ternary systems.

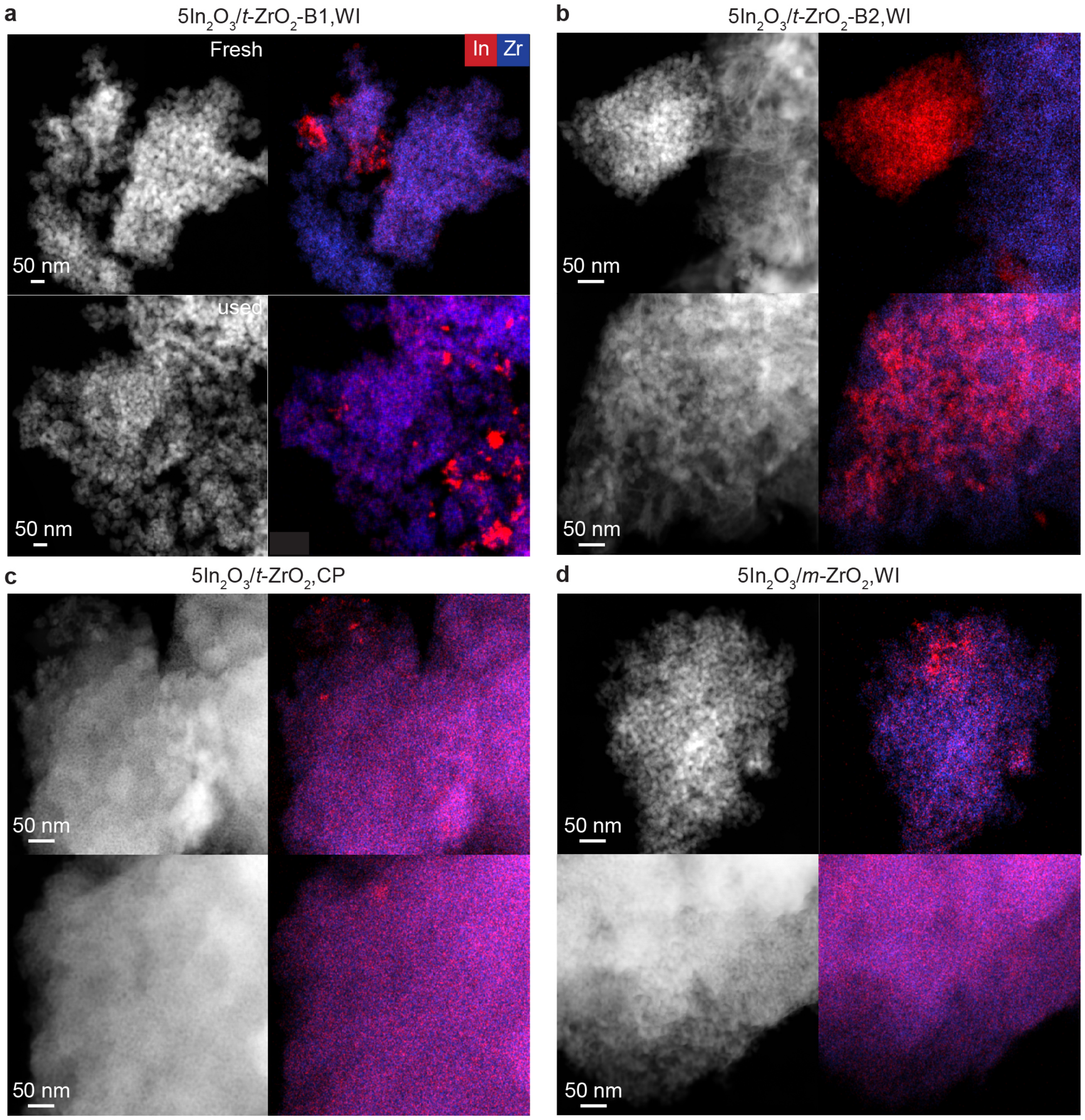

Supplementary Figure 10. Structural organization of ZrO_2-supported In_2O_3 catalysts. HAADF-STEM micrographs with EDX maps of zirconia-supported In_2O_3 catalysts in fresh form and after CO_2 hydrogenation for 50 h. B1 and B2 stand for In_2O_3 wet impregnated onto *t*-ZrO_2 attained by precipitation using different bases (ethylenediamine and NaOH, respectively). Large nanoparticles of In_2O_3 can be clearly seen in **a** and **b** for fresh and used samples, while this phase remains well-dispersed on the support in **c** and **d**. Reaction conditions: $T = 553$ K, $P = 5$ MPa, $H_2/CO_2 = 4$, and $GHSV = 48{,}000$ cm^3 h^{-1} g_{cat}^{-1}.

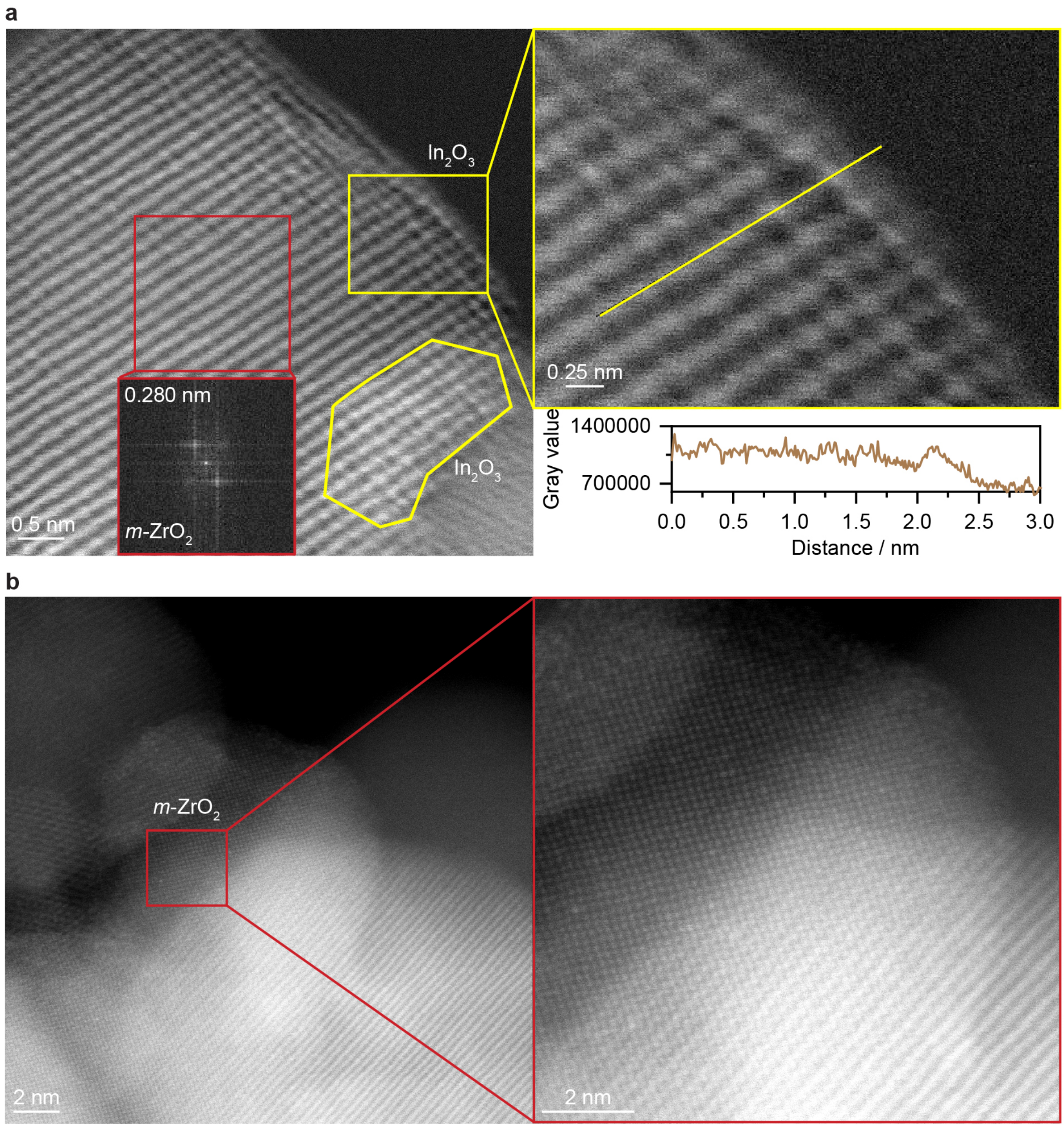

Supplementary Figure 11. Structural organization of the ternary Pd-In_2O_3-ZrO_2 catalyst. (**a,b**) AC-STEM images of 1Pd-5In_2O_3-ZrO_2 catalyst prepared by FSP after CO_2 hydrogenation for 50 h. Insets in **a** depict the lattice distance of *m*-ZrO_2 phase in Fourier-transformed space and some areas containing potential overlayers/islands of In_2O_3 growth, which is evidenced by the line profile. The inset in **b** shows another area at which indium appears to be deposited as adatoms on the surface of ZrO_2. Reaction conditions: $T = 553$ K, $P = 5$ MPa, $H_2/CO_2 = 4$, and $GHSV = 48{,}000$ $cm^3\ h^{-1}\ g_{cat}^{-1}$. The In_2O_3 phase in the catalyst prepared by flame spray pyrolysis appears to wet the surface strongly, leading to a typical thickness of no more than a monolayer. This observation agrees with the high and uniform dispersion of indium over the carrier particles evidenced in elemental maps of the catalyst acquired by energy dispersive X-ray (EDX) spectroscopy (**Figure 4d**). It is also consistent with structures previously observed

for In_2O_3 supported on *monoclinic* zirconia including single and groups of indium oxide adatoms.[1] We note that it is not possible to differentiate between palladium and indium atoms based solely on AC-STEM images. Our assignment takes into account several other factors i) EDX maps indicate a higher dispersion of In on the ZrO_2 carrier than of Pd (**Figure 4d**), ii) as mentioned above isolated adatoms of indium have been previously observed for binary systems comprising In_2O_3 supported on *m*-ZrO_2,[1] and iii) palladium species supported on ZrO_2 by flame spray pyrolysis show a greater tendency to form nanoparticles upon reaction than when the metal is supported on In_2O_3, confirming the less preferential stabilization on ZrO_2.

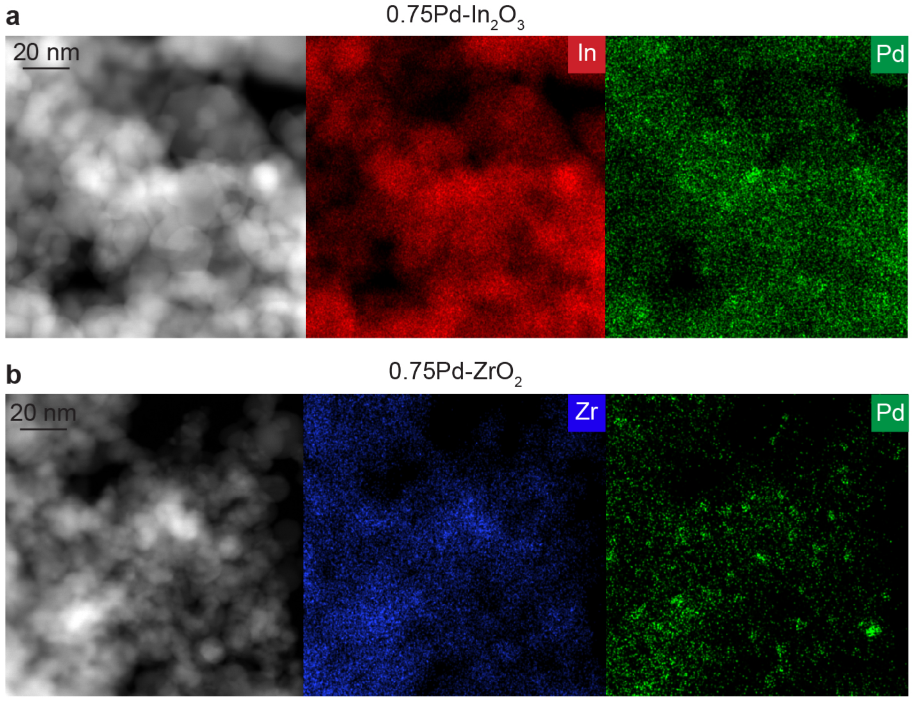

Supplementary Figure 12. Visualization of the palladium distribution on binary catalysts. (**a,b**) HAADF-STEM micrographs with EDX maps of binary Pd-containing systems prepared by FSP after CO_2 hydrogenation for 50 h. Reaction conditions: $T = 553$ K, $P = 5$ MPa, $H_2/CO_2 = 4$, and $GHSV = 48{,}000$ cm^3 h^{-1} g_{cat}^{-1}. Palladium agglomeration is evident in all catalysts, explaining their inferior methanol productivity (see Fig. 2 in the main manuscript).

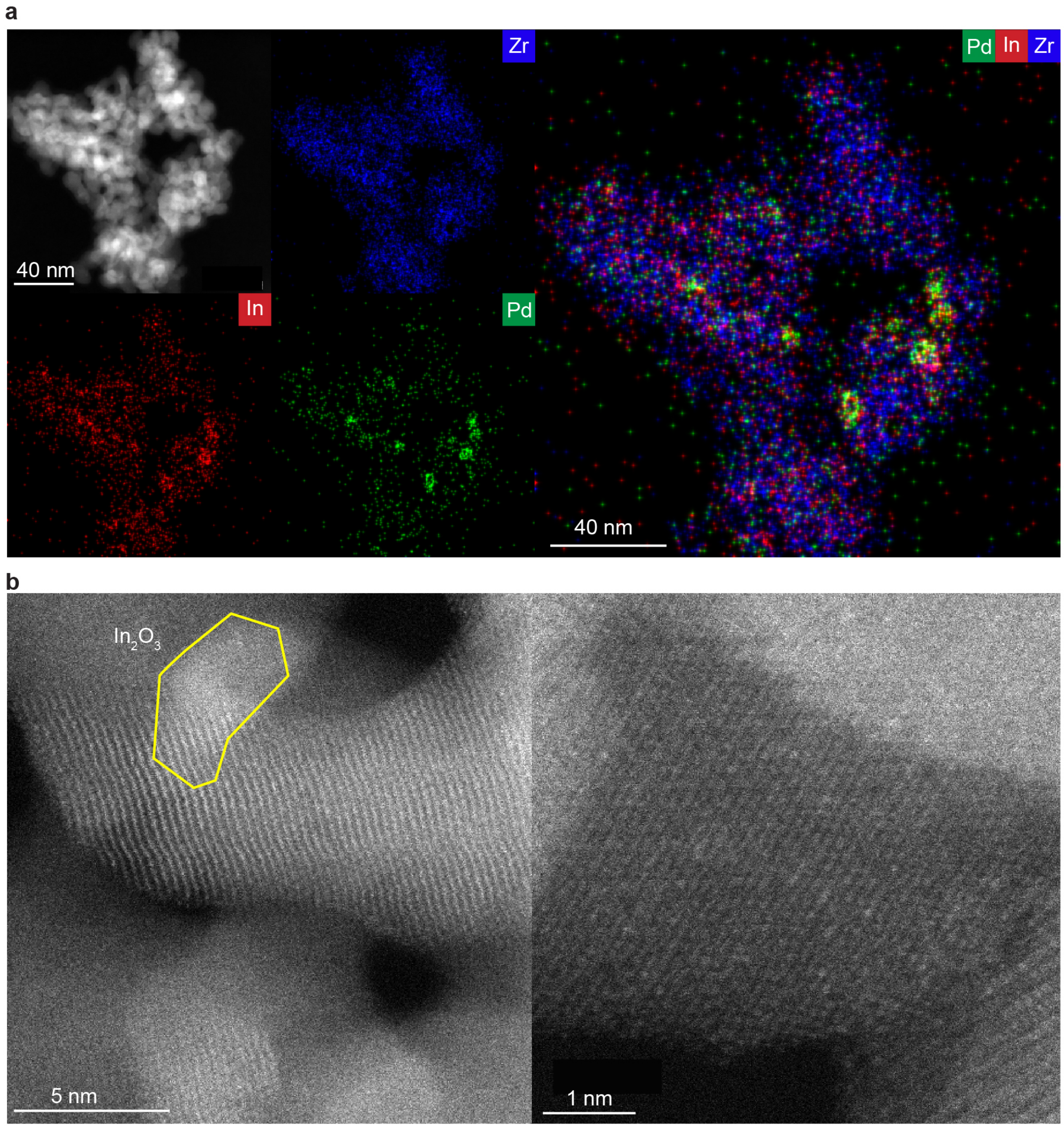

Supplementary Figure 13. Visualization of palladium and indium distribution on the ternary Pd-In_2O_3/ZrO_2 catalyst. (**a**)HAADF-STEM micrograph with corresponding EDX maps and (**b**) AC-STEM images of the ternary Pd-In_2O_3-ZrO_2 system prepared by WI after CO_2 hydrogenation for 50 h. Reaction conditions: $T = 553$ K, $P = 5$ MPa, $H_2/CO_2 = 4$, and $GHSV = 48{,}000$ cm^3 h^{-1} $g_{cat}{}^{-1}$. While In_2O_3 is mainly dispersed as overlayers/islands, palladium tends to agglomerate into large nanoparticles on the catalyst surface, which accounts for its inferior methanol productivity compared to the FSP-made ternary counterpart.

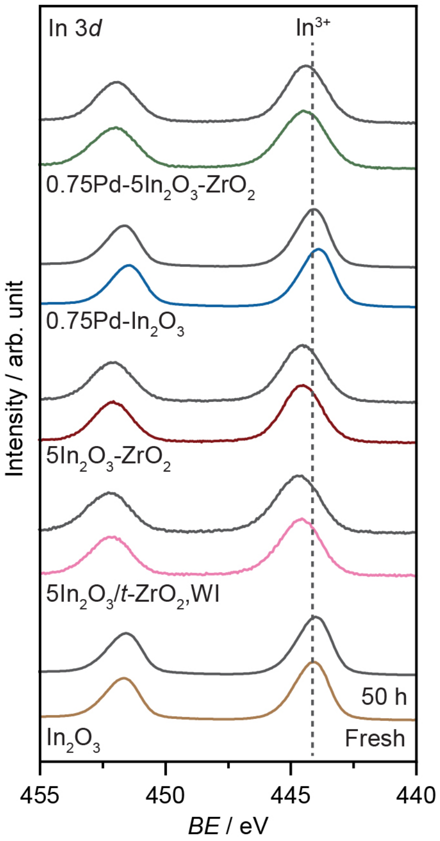

Supplementary Figure 14. Surface indium speciation of In_2O_3-based catalysts. In 3*d* XPS core-level spectra of selected catalysts in fresh and after CO_2 hydrogenation for 50 h. The reference binding energy-*BE* of cationic indium species is indicated by the dashed line. Reaction conditions: $T = 553$ K, $P = 5$ MPa, $H_2/CO_2 = 4$, and $GHSV = 24{,}000$ $cm^3\ h^{-1}\ g_{cat}^{-1}$.

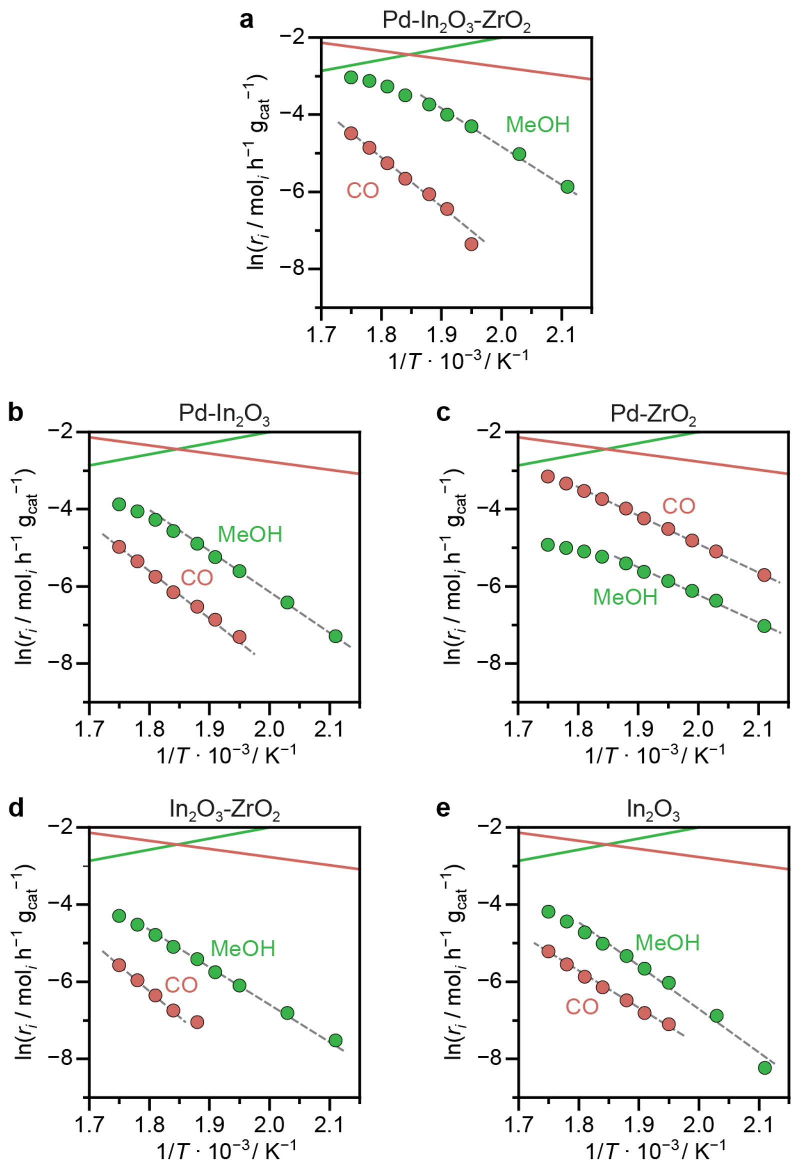

Supplementary Figure 15. Catalytic data used to determine apparent activation energies. (**a-e**) Arrhenius plots used to calculate the apparent activation energies ($E_{a,app}$) for CO_2 hydrogenation to methanol (green) and for the RWGS reaction (red) over selected catalysts prepared by FSP. Solid lines indicate the thermodynamic equilibrium limits of the two independent reactions. Linear regression was performed in the region away from thermodynamic equilibrium as outlined by the dashed lines. Reaction conditions: $P = 5$ MPa, $H_2/CO_2 = 4$, and $GHSV = 48{,}000$ cm^3 h^{-1} g_{cat}^{-1} (except for Pd-In_2O_3-ZrO_2 and Pd-ZrO_2, for which $GHSV = 60{,}000$ cm^3 h^{-1} g_{cat}^{-1}).

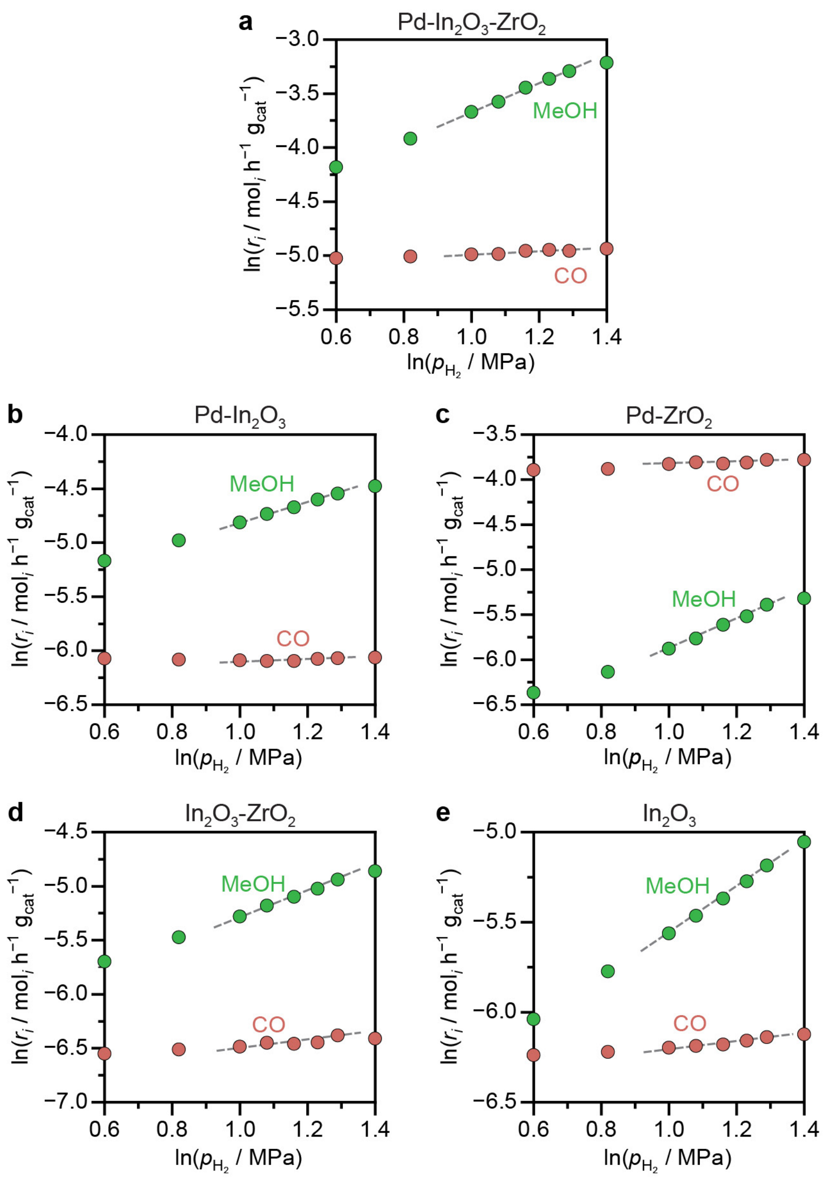

Supplementary Figure 16. Catalytic data used to determine apparent reaction orders with respect to H_2. (a-e) Catalytic data collected at variable partial hydrogen pressures (p_{H_2}) to calculate the apparent reaction orders with respect to hydrogen for CO_2 hydrogenation to methanol (green) and for the RWGS reaction (red) over selected catalysts prepared by FSP. Linear regression was performed in the typical range of H_2/CO_2 ratios of 3-4 as outlined by the dashed lines. Reaction conditions: $T = 553$ K, $P = 5$ MPa, and $GHSV = 48{,}000$ $cm^3\ h^{-1}\ g_{cat}^{-1}$.

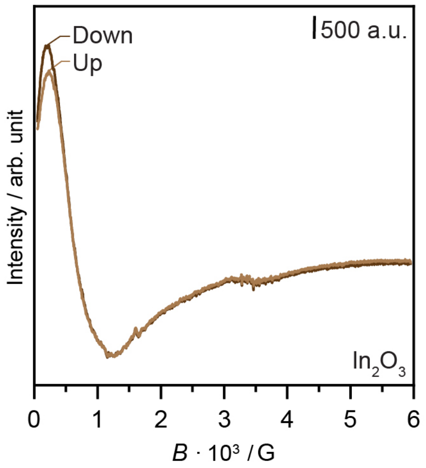

Supplementary Figure 17. cw-EPR analysis of the hysteretic behaviour of the In_2O_3 catalyst. *Ex situ* EPR spectra of In_2O_3 catalyst prepared by FSP in fresh form measured at 303 K, displaying hysteretic behaviour consisting of a difference in intensity and lineshape for upwards and downwards field sweeps.

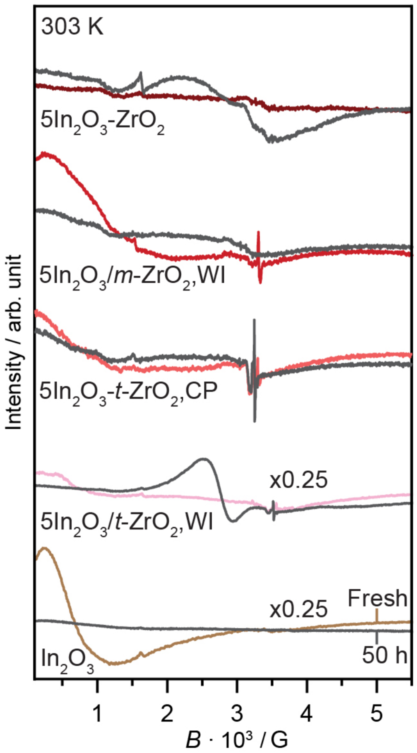

Supplementary Figure 18. Assessment of oxygen vacancies formation on In_2O_3-based catalysts. *Ex situ* EPR spectra of In_2O_3-based catalysts prepared by distinct synthesis methods (WI-wet impregnation, and CP-coprecipitation) in fresh form and after CO_2 hydrogenation for 50 h measured at 303 K. Activation conditions: $T = 553$ K, $P = 5$ MPa, $H_2/CO_2 = 4$, $GHSV = 48{,}000$ cm^3 h^{-1} $g_{cat}{}^{-1}$, and $TOS = 50$ h.

Supplementary References

1. Frei, M. S. *et al.* Role of zirconia in indium oxide-catalyzed CO_2 hydrogenation to methanol. *ACS Catal.* **10**, 1133-1145 (2020).
2. Jung, K. T. & Bell, A. T. The effects of synthesis and pretreatment conditions on the bulk structure and surface properties of zirconia. *J. Mol. Catal. A Chem.* **163**, 27-42 (2000).
3. Tsoukalou, A. *et al.* Operando X-ray absorption spectroscopy identifies a monoclinic ZrO_2:In solid solution as the active phase for the hydrogenation of CO_2 to methanol. *ACS Catal.* **10**, 10060-10067 (2020).
4. Frei, M. S. *et al.* Atomic-scale engineering of indium oxide promotion by palladium for methanol production *via* CO_2 hydrogenation. *Nat. Commun.* **10**, 1-11 (2019).
5. Babu, S. H., Kaleemulla, S., Rao, N. M. & Krishnamoorthi, C. Indium oxide: a transparent, conducting ferromagnetic semiconductor for spintronic applications. *J. Magn. Magn. Mater.* **416**, 66-74 (2016).
6. Coey, J. M. D., Venkatesan, M. & Fitzgerald, C. B. Donor impurity band exchange in dilute ferromagnetic oxides. *Nat. Mater.* **4**, 173-179 (2005).
7. Taniyama, T., Ohta, E. & Sato, T. Observation of 4*d* ferromagnetism in free-standing Pd fine particles. *Europhys. Lett.* **38**, 195-200 (1997).
8. Snider, J. L. *et al.* Revealing the synergy between oxide and alloy phases on the performance of bimetallic In-Pd catalysts for CO_2 hydrogenation to methanol. *ACS Catal.* **9**, 3399-3412 (2019).
9. Yao, L., Shen, X., Pan, Y. & Peng, Z. Synergy between active sites of Cu-In-Zr-O catalyst in CO_2 hydrogenation to methanol. *J. Catal.* **372**, 74-85 (2019).
10. Rui, N. *et al.* CO_2 hydrogenation to methanol over Pd/In_2O_3: effects of Pd and oxygen vacancy. *Appl. Catal. B Environ.* **218**, 488-497 (2017).
11. Wang, J., Sun, K., Jia, X. & Liu, C. CO_2 hydrogenation to methanol over Rh/In_2O_3 catalyst. *Catal. Today* **365**, 341-347 (2021).
12. Sun, K. *et al.* A highly active Pt/In_2O_3 catalyst for CO_2 hydrogenation to methanol with enhanced stability. *Green Chem.* **22**, 5059-5066 (2020).
13. Rui, N. *et al.* Hydrogenation of CO_2 to methanol on a $Au^{\delta+}$-In_2O_{3-x} catalyst. *ACS Catal.* **10**, 11307-11317 (2020).
14. Jia, X., Sun, K., Wang, J., Shen, C. & Liu, C. J. Selective hydrogenation of CO_2 to methanol over Ni/In_2O_3 catalyst. *J. Energy Chem.* **50**, 409-415 (2020).
15. Bavykina, A. *et al.* Turning a methanation Co catalyst into an In-Co methanol producer. *ACS Catal.* **9**, 6910-6918 (2019).

16. Wang, J. *et al.* A highly selective and stable ZnO-ZrO_2 solid solution catalyst for CO_2 hydrogenation to methanol. *Sci. Adv.* **3**, 1-11 (2017).
17. Arena, F. *et al.* Effects of oxide carriers on surface functionality and process performance of the Cu-ZnO system in the synthesis of methanol *via* CO_2 hydrogenation. *J. Catal.* **300**, 141-151 (2013).
18. Bugaev, A. L. *et al.* In situ formation of hydrides and carbides in palladium catalyst: When XANES is better than EXAFS and XRD. *Catal. Today* **283**, 119–126 (2017).
19. Tew, M. W., Miller, J. T. & van Bokhoven, J. A. Particle size effect of hydride formation and surface hydrogen adsorption of nanosized palladium catalysts: L_3 edge *vs* K edge X-ray absorption spectroscopy. *J. Phys. Chem. C* **113**, 15140-15147 (2009).
20. Dann, E. K. *et al.* Structural selectivity of supported Pd nanoparticles for catalytic NH_3 oxidation resolved using combined operando spectroscopy. *Nat. Catal.* **2**, 157-163 (2019).
